# 

**Published:** 1858-02

**Authors:** 


### AMERICAN MEDICAL ASSOCIATION.

The eleventh annual meeting of the American Medical Association will be held in the city of Washington, on Tuesday, May 4, 1858.

The secretaries of all societies, and other bodies entitled to representation in the Association, are requested to forward to the undersigned correct lists of their respective delegations *as soon as they may be appointed; and it is earnestly desired* by the Committee of Arrangements that the appointments be made at as early a period as possible.

The following are extracts from Art. II. of the Constitution:

"Each local Society shall have the privilege of sending to the Association one delegate for every ten of its regular resident members, and one for every additional fraction of more than half this number. The faculty of every regularly constituted Medical College or chartered School of Medicine, shall have the privilege of sending two delegates. The professional staff of every chartered or municipal hospital, containing a hundred patients or more, shall have the privilege of sending two delegates, and every other permanently organized medical institution of good standing shall have the privilege of sending one delegate.

"Delegates representing the medical staffs of the U. S. Army and Navy shall be appointed by the Chiefs of the Army and Navy medical bureaux. The number of delegates so appointed shall be four from the Army medical officers, and an equal number from the Navy medical officers."

ALEX. J. SEMMES, M. D.,
(One of the Secretaries),
Washington, D. C.

**☞ The Medical press of the United States is respectfully requested to copy the foregoing.**

### RECEIPTS ON SUBSCRIPTIONS,

UP TO FEBRUARY 20TH, 1858.

### ILLINOIS.

| | | | |
|---|---|---|---|
| Dr. L. H. Smith, | vol. 1, | 1858, | $2 00 |
| " Thos. Bevan, | " 1, | " | 2 00 |
| " J. L. Hamilton, | " 4, | 1855, | 2 00 |
| " G. W. Hewitt, | " 6, | 1857, | 2 00 |
| " R. Ludlam, | " 1, | 1858, | 2 00 |
| " J. H. Reeder, | " 5&6, | 1856-7, | 5 00 |
| " G. W. Albin, | " 1, | 1858, | 2 00 |
| " A. M. Miller, | " 6, | 1857, | 2 00 |
| " A. S. Haskell, | " 6, | " | 2 00 |
| " T. A. Brown, | " 1, | 1858, | 2 00 |
| " D. D. Thompson, | " 6, | 1857, | 2 00 |
| " Milton Bacon, | " 5&6, | 1856-7, | 4 00 |
| " G. B. Ewing, | " 1, | 1858, | 2 00 |
| " B. W. Hart, | " 6&1, | 1857-8, | 4 00 |
| " W. Pierce, | " 5&6, | 1856-7, | 4 00 |
| " Blake, | " 1, | 1858, | 2 00 |
| " L. B. Brown, | " 1, | " | 2 00 |
| " J. R. Webster, | " 1, | " | 2 00 |
| " Winston Somers, | " 1, | " | 2 00 |

### IOWA.

| | | | |
|---|---|---|---|
| Dr. Ireland, | vol. 6, | 1857, | $2 00 |

### INDIANA.

| | | | |
|---|---|---|---|
| Dr. P. Meyer, | vol. 1, | 1858, | $2 00 |
| " M. Minor, | " 1, | " | 2 00 |
| " J. N. Green, | " 1, | " | 2 00 |
| " B. F. Keith, | " 1, | " | 2 00 |
| " James Evans, | " 1, | " | 2 00 |
| " S. B. Davis, | " 1, | " | 2 00 |
| " A. Heavenridge, | " 1, | " | 2 00 |
| " C. J. Keegan, | " 1, | " | 2 00 |

### WISCONSIN.

| | | | |
|---|---|---|---|
| Dr. E. C. Kimball, | vol. 1, | 1858, | $2 00 |
| " W. M. Rockwell, | " 1, | " | 2 00 |

### MICHIGAN.

| | | | |
|---|---|---|---|
| Dr. A. Foster, | vols. 6&1, | 1857-8, | $2 50 |

### PENNSYLVANIA.

| | | | |
|---|---|---|---|
| Dr. D. B. Elder, | vol. 1, | 1858, | $2 00 |

### MISSOURI.

| | | |
|---|---|---|
| Dr. E. C. Snow, | vols. 1, 2, 3 & 4, | $8 00 |

